# Development and Validation of a Chinese Resilience Scale for Young Children

**DOI:** 10.3390/ijerph20032216

**Published:** 2023-01-26

**Authors:** Zhihong Zuo, Yirui Luo, Juzhe Xi, Weidong Ji

**Affiliations:** 1 Department of Preschool Education, Faculty of Education, East China Normal University, Shanghai 200062, China; 2 Shanghai Key Laboratory of Mental Health and Psychological Crisis, Affiliated Mental Health Center (ECNU), Positive Education China Academy, Juzhe Xi’s Master Workroom of Shanghai School Mental Health Service, School of Psychology and Cognitive Science, East China Normal University, Shanghai 200062, China; 3 Affiliated Mental Health Center (ECNU), Shanghai Changning Mental Health Center, Shanghai 200335, China

**Keywords:** young children, resilience scale, reliability and validity

## Abstract

(1) Background: Resilience research began in the child population as a validity scale to describe children’s psychological wellbeing and ability to cope with negative events, and to some extent, to predict recovery and adaptation when they experience adversity again. In view of the important developmental implications of resilience in young children and the lack of a Chinese children’s resilience scale, this study developed a resilience scale for young Chinese children based on a systematic review of existing international resilience scales and the characteristics of the Chinese cultural background. (2) Methods: The scale was developed by referring to existing scales, expert interviews, item collation and item finalization, developing original items, then deleting and determining items through item analysis, and finally, comparing with existing scales to obtain the internal and external validity of this scale. (3) Results: The results showed that the scale has good measurement properties, internal consistency reliability, and internal and external validity. (4) Conclusions: Through the development and validation of the Resilience Scale for young children in China, the scale can be used to measure the resilience of young children in China.

## 1. Introduction

### 1.1. Concept of Resilience

The understanding of resilience has gone through several stages.

At first, researchers found a phenomenon in which an individual in a stress environment develops the ability to adapt well to face setbacks, difficulties, and dangers in positive ways in that environment. Anthony and Rutter found that a proportion of young children in families with mental illness and facing life adversity could develop relatively high levels of mental health through their own efforts to overcome difficulties in the family and in adversity, respectively [1,2]. In subsequent studies, Werner went on to find that well-adjusted young children, with family social support and positive personality qualities, are capable of facing adversity and difficulties [3]. A review of worldwide research on resilience found that at least 50% of children who encounter adversity grow up to be individuals with good social outcomes [4].

Subsequently, by summarizing this phenomenon, resilience was defined as a psychological quality consisting of multiple factors. As a psychological quality, resilience is considered to be an ability to adapt positively or to maintain or recover mental health despite adversity [5].

However, with the advancement of related research, researchers have found that resilience is more appropriately described as a process of positive dynamic changes in which individuals are able to adapt well despite adversity. In this process, protection factors act simultaneously with risk factors such as adversity and hardship on the individual [6]. Protective factors are divided into three categories: individual level, family level, and community level, which enhance resilience at different but interacting levels [7]. For example, Haase found that the model of the resilience in adolescents consisted of protection, risk, and outcome factors [8]. Brennan found that the main conditions for youth to gain resilience in the face of difficulty and frustration were the presence of social support and the dynamism of the group they were in [9]. Among specific occupational groups, five factors, namely, hope, self-efficacy, control, coping style, and competition, explained 60% of the resilience of the nursing group [10]. Fletcher et al. found that factors related to resilience in the athletic group, such as positive personality and social support, influenced the group’s metacognition and assessment of distressing tasks from the stress they receive [11]. Process theory emphasizes how resilience comes into action during the adaptation process and is more meaningful for interventions that use resilience.

Therefore, in subsequent studies, researchers have focused more on the process theory of resilience. In particular, when talking about resilience in young children, Garmezy suggested that protective and risk factors in resilience act in three different forms in young children coping with difficulties: (1) protective factors act independently of the outcome or suppress risk factors; (2) risk factors act as a stressful stimulus that reduces or enhances the individual’s level of competence; (3) protective factors interact with risk factors, with protective factors acting directly on the outcome and reinforced by risk factors [12]. This process theory of resilience with intertwined factors has provided an important foundation for subsequent research on resilience in young children and is accepted by most researchers. By examining the risk factors and protection factors that constitute resilience, we gain a deeper understanding of the origins of resilience, or in other words, the question of why some of children develop well in spite of adverse circumstances.

### 1.2. Risk Factors and Protection Factors of Young Children

Based on process theory, researchers have focused on the various factors of the resilience process in young children, and more importantly, measures of protective factors can predict the performance and developmental outcomes of young children when they are exposed to risk in the future [13]. However, the study of protective factors is also a gradual transition past risk factors, so this paper provides a brief introduction to both.

First, researchers have focused their attention on high-risk environmental factors of young children. In long-term follow-up studies, maternal pregnancy problems, the family’s economic situation, parental education, unstable family organization, and parental illness have been found to be factors that contribute to children’s high-risk developmental environments [14]. Other studies have also found that a broken family environment, overly strict and spoiled parents, and constant stress have become major factors that lead to children being in a high-risk environment [15]. In addition to the above summary studies of risk factors, in recent years, researchers have focused more on the relationship between a particular risk factor and resilience. For example, a child’s own disorder(s) can also be a risk factor. In studies related to autism spectrum disorders, about 10% to 15% of adults with autism were found to have good developmental outcomes, consistent with the concept of resilience [16]. In addition, in a meta-analytic study of exposure to violence, researchers concluded that children’s exposure to violence is multifaceted in terms of risk factors, including maltreatment, intimate partner violence, and community violence [17]. Likewise, sexual abuse is a high-risk risk factor. The negative effects of sexual abuse on children are consistent across cultures. There are long-term negative effects on children in terms of social, psychological, and sexual functioning [18]. Finally, the specific external environment can also pose a risk factor—unavoidable disasters, for example. Masten took a multi-system perspective to sort out that terrorist attacks, large-scale accidents, natural disasters, technological disasters, and multifaceted disasters that combine natural disasters with technological disasters or human error and negligence are all high-risk factors for children [19]. Specifically, the COVID-19 pandemic has had a negative impact on children in recent years. In the context of the disease epidemic and extended school closures, children’s emotional and social functioning and physical activity levels have decreased [20].

On the other hand, protective factors are also an important component of resilience. In contrast to the dichotomous approach, in which protective factors are divided into child and environmental variables, more researchers tend to divide protective factors into three categories: individual, family, and extra-familial factors [21]. By reviewing previous studies on children’s resilience, researchers have collated those individual factors, mainly including children’s intelligence level, good temperamental level, high social skills, etc.; family factors, including harmonious parental relationship, good family economic status, and good parental qualities; and extra-familial factors, including a good social support system and successful school experiences [22]. Specifically, one study on post-traumatic growth found that higher resilience was associated with greater optimism and positive religious coping [23]. There are also studies on early childhood maltreatment that find participation in extracurricular activities, satisfaction with school and freedom from bullying, and good communication and social skills are important protective factors for resilience [24]. As a summary meta-analysis, Yule, Houston, and Grych found 11 protective factors in cross-sectional studies through 118 studies on resilience over the past 30 years, and self-regulation, family support, school support, and peer support were found to have significant protective effects in longitudinal studies [15].

Through the exploration of risk and protection factors in young children, it would be of significance to provide the reference materials for measuring the level of resilience. This research on the exploration for factors provides the basis for measurement tools not only for the child population alone; it also provides an important foundation for the measurement of resilience for all groups.

### 1.3. Measurement of Resilience

Since the purpose of this paper is to focus on the resilience of young children, the resilience scales that are currently being heavily used are presented through applicable age groups. With this method, some of the problems of the current resilience scale for children can be shown more visually. Here, the resilience scales are divided into two main categories: multi-age and age-specific scales.

The main resilience scales for multi-age groups are the Resilience Scale (RS, by Wagnild in 1993), the Ego-Resiliency Scale (ER89, by Block and Kremen in 1996), and the CD-Resilience Scale (CD-RISC, by Connor and Davidson in 2003).

The original version of the RS was developed by Wagnild, and it contained 25 items scored on a seven-point scale with five components: sense of purpose and meaning, authenticity, equanimity, self-reliance, and perseverance [25], and a reduced version of 14 questions (RS14) was developed later. The original version and the reduced version both have the same age group for the 12-years-and-older group. For the younger age group, Wagnild and Gail developed the Resilience Scale for Children (RS10), which contains the same five sections as the original version of the scale, for the 7–11-year-old group [26].

The Ego-Resiliency Scale (ER89), developed by Block and Kremen in 1996, contains 14 items on a four-point scale that reflects the individuating inventory items in a non-psychotic setting. These items allow for individuals to be flexible and resourceful in response to internal and external stressors [27]. In subsequent use, researchers have developed a shorter, revised version of the ER89-R [28]. In studies using the ER89-R, the results showed full configural invariance for different age groups (16–25 years, 26–40 years, and 40+ years), indicating that the scale demonstrated appropriate testing power across age groups to accurately measure self-resilience as a psychological quality [29].

The CD-Resilience Scale (CD-RISC), which also focuses on the measurement of individual abilities and traits, contains 25 items scored on a five-point scale and involves five factors: personal competence, stress resistance, acceptance of change, sense of control, and religious influence [30]. During the use of the 25-item version of the CD-RISC, Campbell-Sills found that the original scale had some instability with the five factors, and thus developed a 10-item version of the CD-RISC (CD-RISC-10) based on the original scale, which also had good reliability and validity [31]. In practice, the CD-RISC has shown stability and good reliability for secondary school students (13–15 years old), college students and adults (18–30 years old), and older adults (60–75 years old), with adapted results for different age groups [32,33,34]. The scale has been localized by relevant researchers for domestic use in China. For example, the Chinese version of the CD-RISC was revised and translated by Yu and Zhang and showed good reliability during the actual measurement [35]. The Chinese version of the CD-RISC-10 was translated by Ye et al. and tested for reliability and validity, and the results showed its good reliability and validity [36].

The main age-specific resilience scales are the Resilience Scale for adults (RSA, by Friborg, Barlaug, Martinussen, Rosenvinge and Hjemdal in 2005) and the Devereux Early Childhood Assessment for Preschoolers (DECA, by Lebuffe and Naglieri in 1999).

The Resilience Scale for adults (RSA) was developed by Friborg, and it contains 43 items on a seven-point scale with five factors: personal competence, social competence, family cohesion, social support, and personal organization [37]. The target population age group was the adult group over 18 years old, but in the actual test on the elderly group, it was found that although the RSA could identify individuals with lower resilience, the ability to identify individuals with higher levels of resilience was weaker [38]. The Chinese version of the RSA was translated and back-translated several times by Wang et al., and the scale restored the content of the original English scale and conformed to the Chinese language expression [39].

The Devereux Early Childhood Assessment (DECA) was developed by Lebuffe and Naglieri and is divided into two versions: DECA for Infants (DECA-I) and DECA for Toddlers (DECA-T), the former for infants aged 1–18 months and the latter for toddlers aged 18–36 months [13,40]. In 2012, a second version of the DECA for Preschool (DECA-P2) was developed for children aged 3–5 years. This 38-item, five-point scale contains four factors: self-regulation, initiative, attachment and relationship, and behavior problems [41]. Regarding the domestic use of the scale in China, Ji et al. revised the DECA-P2 in Chinese and found that the translated scale had good reliability and validity for assessing a Chinese population of young children [42].

Combining the information of the above scales, there are some differences between different resilience scales for the applicable age groups, and the information of the applicable age groups for each scale is shown in Table 1.

In addition to the above scales, Chinese researchers have also attempted to translate resilience subscales from other scales or to develop locally applicable measurement instruments and validate them in actual testing. For example, Wang et al. used the Student Resilience Survey from the California Healthy Kids Survey and translated it to conduct a study on resilience and social support in young children [43]. Chen and Huang developed the Social Adjustment Scale for secondary school students, and one of the subscales was the Resilience Scale [44]. Hu and Gan developed the Resilience Scale for adolescents for a group of Chinese adolescents [45]. Based on the theory of resilience, Zhang and Lu developed the Adolescent Emotional Resilience Questionnaire to address the emotional characteristics of adolescents [46]. Li and Kwon developed a resilience questionnaire for rural left-behind children group that is applicable to left-behind children [47].

In summary, the review of the age groups to which the resilience scales apply reveals that there is a diversity of resilience scales applicable to the adult population and a lack of resilience scales for young children. In particular, there are no localized resilience scales for young children that are applicable to China.

### 1.4. Research Aims

Through the above summary of domestic and international research on resilience, it appears that there are three main problems in measuring resilience in China.

1. Most of the scales were translated from foreign versions and tested in China. Although most of the researchers made rigorous localization changes in the process of translation, it was not possible to define whether some of the entries on cultural connotations could fully correspond to the Chinese culture. For example, for the item on “God” in the CD-RISC, it is difficult to have a replacement entry covering Chinese culture in the domestic cultural context.
2. The issue of whether the connotations of resilience of individuals in foreign cultures overlap completely with those in Chinese cultures arises. The question of whether there are factors of resilience specific to Chinese culture, which are not measured by corresponding items in foreign measurement instruments, also exists when using translated versions of foreign scales.
3. There are a lack of measurement instruments worldwide that correspond to the early childhood population. Most instruments for measuring resilience are designed for adolescents and adults, and only the DECA-P2 scale is available for measuring resilience in young children. In China, only the revised Chinese version of the DECA-P2 and the resilience questionnaire specifically developed for rural left-behind children are available for the measurement of young children.

In summary, this study aims to develop a localized, culturally inclusive, and universal resilience scale for Chinese young children.

## 2. The Process of Developing the Resilience Scale for Chinese Children

### 2.1. Developing Background

The preschool stage is a critical period in the development of a person’s life. It is a sensitive period for the formation of individual behavior, personality, and ability, and it may also be a sensitive period for the development of mental toughness [48]. In addition, a child’s internal protective factors are measured by determining whether the child has developed satisfactory competencies related to resilience, and if a child scores relatively low on the overall internal protective factors, he or she may be at risk and needs intervention [13]. For example, DECA selected three aspects related to social-emotional competence as an indicator of psychological resilience, which helps to promote emotion recognition and emotion management for the development of empathy, decision-making ability, and good social relationships [49,50]. Therefore, this paper explores personal factors in the resilience of young children, with reference to the way existing resilience scales for young children have been developed as a basis for scale formation.

### 2.2. Developing Process

First, parents of young children were interviewed, 20 in total, and information saturation was reached by asking respondents five questions, the core one of which asked respondents to “describe a process in which their child encountered difficulties or challenges”. The remaining four questions were designed to help respondents extract memories, such as “What did your child do to cope with difficulties or challenges?” (What did they say, how did they do it, how did they say it?). The interview time was 20 min per person.

Then, the interview materials were transcribed in a timely manner to ensure accuracy, reflect on the problems in the interviews, revise the interview outline in a timely manner, and conduct a category analysis using content coding. The interview information was categorized into items, i.e., the core meaning of the interviewees’ responses was extracted, the sentences were made complete, written, and concise, and similar items were combined.

Finally, six experts were invited, including two in developmental psychology, two in preschool education, and two in clinical and counseling psychology. The items with objections were discussed, and 70 items were set in a response format, presenting the 70 items in a five-point Likert scale, i.e., 1 (never), 2 (rarely), 3 (sometimes), 4 (often), and 5 (very often). Higher scores indicated greater level of resilience.

## 3. Exploratory Factor Analysis of the Resilience Scale for Chinese Children

### 3.1. Participants

Data were obtained from 499 young children, including 249 males, 248 females, and 2 with missing gender information. The informants were 347 mothers, 135 fathers, 15 grandparents, and 2 missing informants. The mean age of the children was 4.43 ± 1.03.

### 3.2. Procedure

The scale uses the same measurement format as the DECA-P2 and can be scored by teachers or their primary caregivers who are in close contact with young children. The primary caregivers in this study completed the questionnaire on a voluntary basis, and data analysis was completed using SPSS 26.0 statistical software.

### 3.3. Results

The first step was to delete 15 items based on item discrimination <0.2, that is, a total score of 27% higher or lower, and the difference between the mean groups of each item were divided by the group distance of the five-point scale. In the second step, EFA and the gravel plot showed that four factors was an inflection point, suggesting that the four factors were appropriate for an eigenvalue >1. Then, deleting the loading low with cross-loading items to the last remaining 20 items, the number of question items was 7, 5, 4, 4, and cumulative variance explained 62.821%, KMO = 0.924, Bartlett’s Test of Sphericity test x^2^ = 3291.65, *df* = 190, *p* < 0.001. Finally, the number of items under each dimension was determined to be four based on item discrimination again and balancing the number of items for each factor, for a total of 16 items with a cumulative variance explaining 64.493%, KMO = 0.901, Bartlett’s Test of Sphericity test x^2^ = 2220.43, *df* = 190, *p* < 0.001. The scale structure and factor loadings for the 16 items are shown in Table 2.

The 16-item Resilience Scale for Chinese Children was divided into four factors, with 64.5% total variance explained and 33.45%, 10a.96%, 9.19%, and 7.87% variance explained by individual factors, respectively. The four factors were named according to their significance, and the internal consistency coefficient was calculated for each factor: 1—social and agreeable, *α* = 0.83; 2—emotional stability, *α* = 0.83; 3—focused engagement, *α* = 0.72; 4—self-caring, *α* = 0.71. The four factors reflect the child’s social, emotional, cognitive, and behavioral abilities to help him or her face difficulties in the face of adversity, with a total scale *α* = 0.87.

### 3.4. Discussion

The main purpose of the exploratory factor analysis of the original scale was to reduce the total number of items on the scale in order to keep the scale at a reasonable level of usefulness and difficulty when measured with young children. As shown in Table 2, the scale contains only 16 items, and the exploratory factor analysis was repeated several times so that the factor loadings of the 16 retained items ranged from 0.56 to 0.82, and the number of items was uniform across the four dimensions to be tested in terms of structural dimensions. The cumulative variance interpretation was also at the appropriate level, and there was a high level of content consistency.

## 4. Validation Factor Analysis of the Resilience Scale for Chinese Children

### 4.1. Participants

There were 374 young children, including 202 males and 172 females. The age distribution was between 2 and 6 years old (*M* = 4.83, *SD* = 1.03). There were 283 mothers (75.7%), whose education distribution was 35 junior high school and below, 31 high school/junior high school, 52 college/high school, 117 university, 34 master’s degree students, and 14 doctoral degree students; 91 fathers (24.3%), whose education distribution was 3 junior high school and below, 10 high school/junior high school, 13 college/high school, 39 university, 19 master’s degree students, and 7 doctoral degree students.

### 4.2. Procedure

This analysis sample was scored by the primary caregiver using the same measurement format as the initial test, and data analysis was completed using SPSS AMOS 26.0 statistical software.

### 4.3. Results

**Figure 1:**
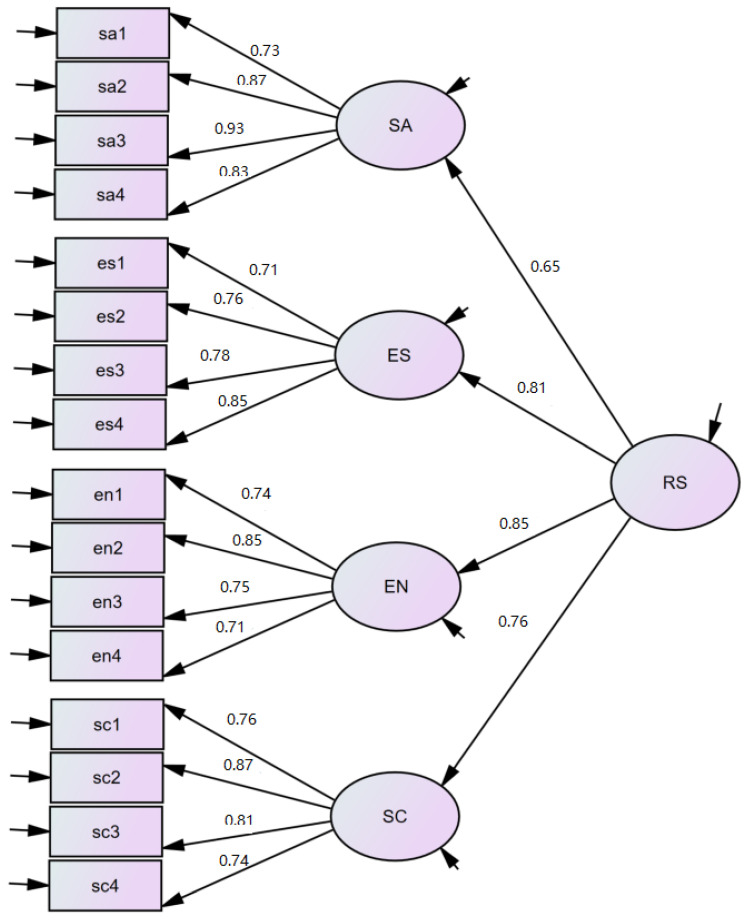
Factor structure model of the Resilience Scale for Chinese Children. (SA: social and agreeable; ES: emotional stability; EN: engagement; SC: self-caring; RS: Resilience Scale).

The path map obtained by the validation factor analysis is shown in Figure 1. The corresponding fit indices are shown in Table 3.

### 4.4. Discussion

As can be seen in Figure 1, the specific items tested in the four areas maintained relatively good levels of path coefficients, which ranged from 0.71 to 0.93. The four factors tested also showed path coefficients between 0.65 and 0.85 for the resilience. For the model obtained from this validation factor analysis, the more commonly used fit indexes were selected for reporting, and in the fit indexes presented in Table 3, the model fit was at an acceptable moderate level: *X²/df* between 1 and 5, TLI > 0.90, CFI > 0.90, SRMR = 0.05, and RMSEA > 0.05 [48].

## 5. Internal, Structural, and External Validity Tests of the Resilience Scale for Chinese Children

Internal and structural validity: In this study, internal consistency was used as the internal validity index of this scale, and through the validation of the EFA process above, the Cronbach’s *α* for the 16 items of this scale was 0.87, indicating that this scale has high internal consistency, which means it has acceptable internal validity [49]. Regarding the structural validity of a scale, it is also often characterized by the fit indexes in EFA, in addition to examining the degree of correlation between the factors in the scale, which is also a way to examine structural validity [45]. Given that the fit indexes of the scale have been described above, the degree of correlation between factors within the scale is validated here to complement the performance of the scale’s structural validity. External validity: This study examined the criterion-related validity of the Resilience Scale for Chinese Children to measure the external validity. The criteria contain four early childhood measurement scales: the Preschool Behaviour and Emotional Rating Scale (PreBERS), the Early School Behavior Scale (ESBS), the Child Resilience Measure-Revised Person Most Knowledgeable version (PMK-CYRM-R), and the Face Scale (FS). These four scales cover three aspects of physical, psychological, and social functioning of young children, which can better reflect the overall level of functioning of young children.

PreBERS consists of 42 items and contains four dimensions, namely, emotional regulation, school readiness, social confidence, and family involvement. Each subscale consists of 13 items and is scored on a four-point Likert scale from 0–3, with higher total scores indicating better adjustment [50]. According to a study conducted by Chinese scholars Hua and Zhou, item 17 of the original scale, “comparable to children of the same age in terms of hygiene skills” and item 24, “good interaction with siblings in the family”, were not applicable to Chinese preschoolers [51]. Therefore, these 2 items were deleted, leaving 40 items. The scale was reduced to take into account the length of the test, and the four dimensions were retained and analyzed for factor structure: four questions on emotion regulation, *α* = 0.71, four questions on school readiness, *α* = 0.88, three questions on social confidence, *α* = 0.75, and four questions on family involvement, *α* = 0.93, for a total of 15 questions. The total summary scale had *α* = 0.86 and explained 70.45% of the total variance. Factor loadings for each question were at the appropriate level.

ESBS contained 46 entries divided into three dimensions: the Competence scale, 19 questions; the Anxiety scale, 18 questions; and the Conduct Problems scale, 9 questions. The Competence scale contains four domains: self-regulation, prosocial skills, tension management, and social adjustment. The Anxiety scale and the Conduct problem scale correspond to children’s internalizing and externalizing behaviors, respectively [52]. In this study, the original scales were abbreviated by retaining five entries on each subscale to form a simplified 15-question scale for use. Factor structure analysis of the simplified scales yielded Competence scale *α* = 0.83, Anxiety scale *α* = 0.73, and Conduct Problems scale *α* = 0.73.

PMK-CYRM-R contains 17 entries divided into two subscales, the intra/interpersonal and caregiver resilience subscales. A five-point Likert scale from 1–5 was used for scoring, with higher total scores representing higher levels of resilience [53]. This study used one of the internal/interpersonal subscales as an indicator of resilience with 10 questions and Cronbach’s *α* = 0.88. A three-point Likert scale from 1–3 was used for scoring.

FS consists of seven faces, 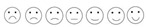, which are identified in order from left to right on a scale of 1–7. Higher scores represent higher levels of happiness for the individual [54].

### 5.1. Participants and Procedures

PreBERS in internal validity tests and external validity tests used the same subject groups as in the validation factor analysis (*N* = 374).

The subjects group used for ESBS, PMK-CYRM-R, and FS in internal validity tests consisted of 1082 children, including 602 males and 480 females. The grade distribution was between nursery and senior classes (184 children (17%) in nursery classes, 173 children (16%) in junior classes, 360 children (33.3%) in middle classes, and 365 children (33.7%) in senior classes). There were 513 (47.4%) fathers and 569 (52.6%) mothers who provided information.

All scales were completed by the primary caregiver, and the Resilience Scale for Chinese Children was then continued to be administered to this sample. Data analysis was completed using SPSS 26.0 statistical software.

### 5.2. Results and Discussion

The structural validity of the scale was analyzed by examining the degree of correlation within the scale. As shown in Table 4, the two-by-two correlation coefficients for the four dimensions ranged from 0.45 to 0.58, a moderately significant correlation, indicating that the factors were consistent in direction but distinct and not substitutable for each other. The correlations between the factors and the overall scores ranged from 0.77 to 0.81, indicating that the factors were internally consistent with the overall measure.

The external validity of the scale was analyzed by examining the correlation between the scores on the four factors of the scale and those on the test questions of other related scales. As shown in Table 5, the scale presented in this study showed significant positive correlations in those dimensions related to protective factors, with correlation coefficients ranging from 0.22 to 0.63. This proves that there is an acceptable convergent validity between the dimensions of this scale and the dimensions of protective factors. In contrast, significant negative correlations were demonstrated on those dimensions related to risk factors, that is, the Anxiety and Conduct Problems in the ESBS, with correlation coefficients ranging from −0.36 to −0.17, demonstrating the existence of acceptable discriminant validity between the dimensions of this scale and those related to risk factors.

## 6. General Discussion

### 6.1. Reliability and Validity of the Scale

This study started with the development of a Chinese localized psychological scale for young children, referred to relevant resilience scales for young children at both domestic and international levels, and through expert interviews, item collation, and item finalization, developed an original resilience scale containing 70 items for use with the Chinese young child population.

On the original basis of 70 items, the original 70 items were reduced by item analysis of 499 valid questionnaires, and retained to 16 items, containing four factors, namely, social and agreeable, emotional stability, engagement, and self-caring, each containing four items, which were basically consistent with the structure and classification of the scale when it was prepared. The factor model and internal validity of the scale were then analyzed by factor analysis of 374 valid questionnaires, a preliminary external validity analysis was conducted, and the results showed a good level of fit and internal consistency. In terms of the number of entries, compared with other resilience scales for young children, this scale contains fewer entries, which reduces the difficulty of measurement. Among the more widely used early childhood scales, most have more than 30 test items, such as the DECA-P2 scale with 38 items, the PreBERS scale with 42 items, and the Social Competence and Behavior Evaluation Scale-30 (SCBE-30) with 30 items [55]. In addition, in most of the resilience scales, there is some variation in the number of items under each factor, such as 43 items in the five-factor RSA scale and 38 items in the five-factor DECA-P2, the number of items measured under each factor is not uniform, and there may be a problem of weighting differences in the specific expression of some of the resilience connotations. In terms of factor connotations, most of the other early childhood resilience scales focus on social-emotional competencies and lack measures of social-behavioral competencies, whereas scales with social-behavioral measures lack social-emotional competencies. It can be argued that few scales in previous early childhood resilience scales have measured social-emotional and social-behavioral competencies in a balanced manner. The present scale combines social-emotional and social-behavioral competencies, balancing the weights of both. Finally, an in-depth analysis of the external validity of the present scale was continued by examining the degree of correlation between the present scale and other related scales through 1082 composite valid questionnaires. When examining external validity, if the correlation coefficients and significance are too high, there is an inevitable risk that the structure of the scale developed and the psychological qualities measured in this study will be too similar to scales that already exist. The results of the analysis of the present scale with other scales showed that the present scale was significantly correlated with each item in other scales, and the correlation coefficients were at a moderate level. Such results suggest that the present scale does not duplicate to some degree the existing scales to a great extent. Among the other scales used in this study, to a certain extent, they do not serve as alternative scales for measuring resilience in young children.

### 6.2. Comparison with Extant Child Scales

Resilience is an extremely rich concept in terms of connotations. As mentioned above, the concept of resilience can be described by different researchers from different theoretical fundamentals. From the extant early childhood scales, the PMK-CYRM-R and DECA-P2 are very widely used scales, with the PMK-CYRM-R containing internal and external interpersonal relationships and caregiver resilience, and the DECA-P2 containing initiative, self-regulation, attachment/relationship, and behavior problems. The factors derived from this study, however, contain social and agreeable, emotional stability, engagement, and self-caring, four factors that both overlap and differ from other scales measuring resilience in young children. The four factors of this study include the internal and external interpersonal relationships in the PMK-CYRM-R, but caregiver resilience, in its connotative nature of resilience, is not a quality possessed by young children themselves, so the scale developed for this study does not include this one dimension. In terms of the specific connotations of the factors, social and agreeable is somewhat related to initiative and attachment/relationship, emotional stability is somewhat related to self-regulation, and self-caring is somewhat related to behavior problems. Meanwhile, engagement is unique to this scale; although engagement has some similarities with initiative, initiative does not describe the ability of young children to be active and focused on things to some extent. Such speculation is based on the possible inappropriateness of the subjects in Chinese culture administered by the two extant foreign language scales. Therefore, the nature of the connotation of resilience and the cross-cultural differences remain to be further investigated. In addition, this scale has fewer questions and the same number of questions on each dimension compared to the PMK-CYRM-R and DECA, which is more conducive to administering and balancing the weights under each dimension.

### 6.3. Limitations of the Study

This study suffers from the problem of a small sample size and the fact that the subjects measured in each phase are in different groups. The problem with the sample size is that for the sample size of the whole study, when the validation factor analysis was conducted, the sample size used was somewhat small compared to the sample sizes used in the other analysis stages. However, by reviewing the data requirements for factor analysis, the current sample size for factor analysis is generally considered to be a minimum of 50 and should be more than five times the number of variables [56], and the sample size for each stage in this report meets the requirements for conducting factor analysis and satisfies the measurement science requirements. The problem with the measured groups is that the samples of the administered groups for the last three of these scales (Child Resilience Measure-Revised Person Most Knowledgeable version, PMK-CYRM-R, the Early School Behavior Scale, ESBS, and the Face Scale, FS) were consistent in the process of external validity validation. However, the first scale (Preschool Behaviour and Emotional Rating Scale, PreBERS) used a sample of the other groups, which was smaller than the sample of the former groups. In addition to this, this study used different sample groups in the EFA, VFA, and validity validation phases, and although such an approach exists in the development of other resilience scales [45], and some previous researchers have found that full validation of the scale requires repeated analyses of samples that are heterogeneous in terms of structure, homogeneous in terms of other characteristics, and demographically similar to the target population [57,58], whether there are risks involved will need to be verified in subsequent studies.

## 7. Conclusions

This scale can be used to measure the resilience of young children in their native Chinese cultural environment, thus advancing research related to the psychological health and development of young children in China. It can also be used as a reference indicator in early childhood education to provide some description of the level of psychological wellbeing of young children in China. In the present study, it was found that the social and agreeable factor accounted for the highest proportion of the explained variance, which also suggests that the social and agreeable element of young children in early childhood education can contribute to their level of mental health.

In future research, we can focus on the extension of the use of this scale, and we can try to change the subjects from the child’s caregiver to the teacher, who is the main observer of the child’s life and learning and has more comprehensive information than the child’s caregiver, to a certain extent, so as to develop a teacher’s version of this resilience scale, and also to avoid to the child’s caregiver’s influence in answering the questions, avoiding the subject effect in the response process. The teacher version is also more convenient than the adopter version in terms of administration, and it is easier to track the resilience of young children and observe the predictive ability of the scale. However, it is important to note that resilience is a broad concept that varies across cultures, and this variation is reflected in the different dimensions and items presented in the scales. The present scale was developed with more of an attempt to expand and validate the items based on the existing scales. This experimental development based on a non-native scale is more likely to require more relevant studies to use this scale for repeated validation of its usefulness.

## Figures and Tables

**Table 1:** Age groups to which different resilience scales were applied.

Scale Name	Applicable Age					
Resilience Scale, RS					12+ years old	
RS14					12+ years old	
RS10				7–11 years old		
Ego-Resiliency Scale, ER89						16–40+ years old
ER89-R						16–40+ years old
CD-Resilience Scale, CD-RISC					13–75+ years old	
CD-RISC-10					13–75+ years old	
Resilience Scale for adults, RSA						18+ years old
Devereux Early Childhood Assessment, DECA						
DECA-I	1–18 months old					
DECA-T		18–36 months old				
DECA-P2			3–5 years old			

**Table 2:** Factor structure of the Resilience Scale for Chinese Children.

Title number	Factor			
	1	2	3	4
Q64	0.78			
Q66	0.81			
Q54	0.79			
Q16	0.74			
Q56		0.82		
Q6		0.74		
Q57		0.76		
Q45		0.71		
Q14			0.76	
Q13			0.76	
Q15			0.75	
Q43			0.56	
Q51				0.76
Q35				0.68
Q37				0.67
Q52				0.66

**Table 3:** Fit indexes for validated factor analysis of the Resilience Scale for Chinese Children.

*X²*	*df*	*X²/df*	TLI	CFI	SRMR	RMSEA
327.816	100	3.278	0.926	0.938	0.050	0.078

**Table 4:** Correlation matrix of the total score and subscales of the Resilience Scale for Chinese Children (*N* = 374).

	SA	ES	EN	SC
ES	0.50 **			
EN	0.47 **	0.58 **		
SC	0.45 **	0.53 **	0.57 **	
Total Score	0.77 **	0.82 **	0.82 **	0.79 **

** *p* < 0.001.

**Table 5:** Correlation coefficients between the four early childhood measurement scales and the Resilience Scale for Chinese Children in terms of total and subscale scores.

		The Resilience Scale for Chinese Children				
		Total Score	SA	ES	EN	SC
PreBERS (*N* = 374)	Total Score	0.59 **	0.43 **	0.49 **	0.52 **	0.46 **
	Emotional Regulation	0.33 **	0.18 **	0.42 **	0.24 **	0.22 **
	School Readiness	0.57 **	0.37 **	0.44 **	0.56 **	0.47 **
	Social Confidence	0.41 **	0.46 **	0.26 **	0.32 **	0.28 **
	Family Involvement	0.39 **	0.29 **	0.23 **	0.36 **	0.36 **
ESBS (*N* = 1082)	Competence	0.57 **	0.46 **	0.46 **	0.50 **	0.52 **
	Anxiety	−0.25 **	−0.26 **	−0.18 **	−0.17 **	−0.23 **
	Conduct Problems	−0.25 **	−0.07 *	−0.36 **	−0.18 **	−0.24 **
PMK-CYRM-R (*N* = 1082)		0.63 **	0.55 **	0.50 **	0.54 **	0.55 **
FS (*N* = 1082)		0.30 **	0.29 **	0.23 **	0.27 **	0.25 **

*p < 0.05, ** *p* < 0.001.

## Data Availability

The data presented in this study are available on request from the corresponding author. The data are not publicly available due to privacy.
